# Soluble guanylyl cyclase regulates skeletal muscle fiber type plasticity, fatigue resistance and whole body insulin resistance

**DOI:** 10.1186/2050-6511-16-S1-A8

**Published:** 2015-09-02

**Authors:** Younghye Moon, Jordan Balke, Michael Siegel, Peter Brouckaert, Emmanuel S Buys, David Marcinek, Justin Percival

**Affiliations:** 1 Department of Molecular and Cellular Pharmacology, University of Miami Miller School of Medicine, Miami, FL 33136, USA; 2 Department of Bioengineering, University of Washington, Seattle, WA 98109, USA; 3 Department for Molecular Biomedical Research and Biomedical Molecular Biology, Ghent University, B-9052 Ghent, Belgium; 4 Department of Anesthesia, Critical Care and Pain Medicine, Anesthesia Center for Critical Care Research, Massachusetts General Hospital, Boston, MA 02114, USA; 5 Department of Radiology, University of Washington, Seattle, WA 98109, USA

## 

Pathogenic defects in NO-cGMP signaling drive skeletal muscle dysfunction in Duchenne muscular dystrophy (DMD). These defects arise from decreased nNOS and guanylyl cyclase (GC) activity and loss of spatial control of NO production. Therapeutics such as sildenafil that amplify NO-cGMP signaling reduce skeletal muscle dysfunction in DMD patients and mouse models of DMD making GC and cGMP attractive therapeutic targets. However, GC and cGMP functions in skeletal muscle are poorly defined hindering therapy development. To remove this barrier, we investigated the functions of GC in skeletal muscle.

We report that α1β1 soluble GC (sGC) is the primary cGMP source in skeletal muscle. α1β1 sGC expression was greater in oxidative muscles suggesting a greater cGMP synthesis capacity and muscle-specific differences in sGC function. Interestingly, sGC activity exhibited partial nNOS dependence. Analyses of sGC subcellular localization revealed a pool of α1β1 at the *cis*-Golgi complex in muscle cells. α1β1 and α2β1 sGC localized to the microvasculature. Muscles lacking a functional α1 sGC subunit (α1β1 sGC deficient) exhibited reduced fatigue resistance and normal hypertrophic growth. Surprisingly, α1β1 sGC deficiency had no impact on mitochondrial content suggesting that mitochondrial density may not be as tightly regulated by cGMP as previously thought. α1β1 sGC deficiency had a modest effect on mitochondrial ATP synthesis. Also, loss of α1β1 sGC triggered a type IIA to type IIX fiber shift. Although this shift was unlikely to significantly enhance fatigability, it may impact insulin metabolism because type IIX fiber content positively correlates with insulin insensitivity. Indeed, α1β1 sGC null mice exhibited gender-specific defects in whole body insulin sensitivity consistent with reports of gender-specific effects of cGMP on metabolism but normal glucose tolerance indicating compensatory changes in glucose metabolism.

In summary, these findings argue that NO signaling through sGC plays important roles in muscle fatigue and fiber type specification and suggest sGC as a target for mitigating skeletal muscle fatigue in DMD. These data also suggest that disruption of NO-cGMP signaling may contribute to the poorly understood metabolic defects in DMD patients. By showing that reductions in cGMP synthesis promote muscle fatigue, type IIX content and insulin resistance, these data suggest new insights as to how decreases in cGMP may contribute to the development of metabolic dysfunction and disease, particularly over time. Importantly, these results suggest sGC as a potential target for mitigating insulin resistance.

